# Co-designing laryngectomy accessible information to support the development of an education and training package for community healthcare professionals: experiences and reflections from multiple stakeholders

**DOI:** 10.1186/s40900-026-00865-w

**Published:** 2026-06-03

**Authors:** Laura-Jayne Watson, Wendy Wadey, Malcolm Babb, Domenic Risi, Dan Waterston, Scott Barrass, Linda Sharp, David W. Hamilton, Vicky Thornton, Joanne M. Patterson

**Affiliations:** 1 South Tyneside and Sunderland NHS Foundation Trust, Sunderland, UK; 2 Liverpool University, Liverpool, UK; 3 South Tyneside and Sunderland, Sunderland, UK; 4 National Association of Laryngectomy Clubs, Chesterfield, UK; 5 National Association of Laryngectomy Clubs, Edinburgh, UK; 6 Freelance Illustrator, Sunderland, UK; 7 Creo Comms, Sunderland, UK; 8 Newcastle University, Newcastle-upon-Tyne, UK

**Keywords:** Laryngectomy, Animation, Infographic, Collaboration, Co-design, Education, Training

## Abstract

**Background:**

Laryngectomy is complete surgical removal of the voice-box, resulting in permanent and life-changing functional and physical alterations for people and their families. There are approximately 460 people who have a laryngectomy every year in England. After laryngectomy people need ongoing support at home following hospital discharge. However, community-based healthcare professionals often lack specialist laryngectomy skills and have limited access to laryngectomy education or training. Current education and training resources for community-based professionals are difficult to navigate and there is lack of consistency in the quality and content of the information provided.

**Main body:**

To begin to address this gap, we brought together people with a laryngectomy, healthcare professionals, a designer and creative agency, to develop a ‘back to basics’ animation and infographic for laryngectomy. We report the outline of the concept and iterative, collaborative, development of the animation, as well as personal reflections from key stakeholders involved in the project. The future direction and use of the animation and infographic is also explored.

**Conclusion:**

This project demonstrates value of collaborative working in the development of an animation and infographic; the basis of the idea being born out of identified gaps in the research. Moving forward, the animation and infographic will be used in co design workshops to shape a community focused education and training package for healthcare professionals supporting people with a laryngectomy. Following this, the animation and infographic will be shared more broadly with healthcare professionals, individuals living with a laryngectomy, and the wider public. These resources have the potential to enhance clinical practice, empower people living with a laryngectomy with clear and accessible information, and raise awareness within the community. Ultimately, this work aims to support more informed, confident, and compassionate care for those living with a laryngectomy.

## Abstract (plain language summary)

Laryngeal cancer (cancer of the voice-box) is one of the most common head and neck cancers (HNC) in the UK. 1,771 people were diagnosed with laryngeal cancer in 2019. Approximately half of people with advanced laryngeal cancers have surgery, including laryngectomy (surgical removal of the voice-box). Laryngectomy is a life-changing operation for people and their families. It results in permanent changes to how people speak, breathe, eat and drink. It also changes the person’s appearance. Once at home after their surgery, people with a laryngectomy need support from healthcare professionals. This is to help them cope and (re-)adjust to life. Healthcare at home is often provided by general services e.g., GPs. These professionals may not have specialist laryngectomy skills. Recent research has also shown a gap in professional education and training. This gap is particularly for community healthcare professionals. There is a need for patients and healthcare professionals to develop education together. Doing this may help to improve the experience of community care for people with a laryngectomy, as well as improving the confidence of community healthcare professionals. This work describes the steps taken to try and begin to close this gap. This has been done through developing an education resource - animation and infographic. Patients, healthcare professionals, a designer and creative agency worked together to develop the animation and infographic. We explain the initial idea and development of the animation and infographic and plans for future use. We also reflect on joint working with many stakeholders and the impact this has had on all involved.

## Background

### What is the problem?

Laryngectomy is complete surgical removal of the voice-box, most often undertaken for treatment of advanced laryngeal cancer [1]. This results in permanent changes to people’s breathing, communication, swallowing and appearance, which is life-changing for them and their families [2]. Healthcare interventions for people with a laryngectomy are complex, necessitating input from multiple organisations across tertiary, primary and community care to deliver long-term rehabilitation. The UK has recently published the NHS long-term plan [3] in which it aims to provide integrated healthcare services, promoting “better support and properly joined-up care at the right time in the optimal care setting.” However, the reality is often different due to funding constraints, workforce shortages and organisational pressures which are impeding progress towards integrated care [4]. UK head and neck cancer services are centralised, meaning that people are referred from wide geographical areas to specialist units for their care. As such, input from laryngectomy specialist healthcare professionals for patients in the community remains inconsistent, with only a small number of regions offering structured community-based laryngectomy services. Primary and community care teams therefore are under significant pressure to provide these services, but often have limited time, busy caseloads and lack the necessary specialist skills. This latter limitation is likely due, in part, to the limited community-specific laryngectomy education or training [5].

### Available education and training resources

There are various options through which laryngectomy education and training are currently delivered. In the UK, NHS organisations offer internal training programmes, however very few make these publicly available or publish their content, limiting the opportunity to standardise the approach to training [6]. This has implications for post-graduate training opportunities and also highlights the gap in undergraduate training for healthcare professionals. The UK also has two national organisations offering laryngectomy education and training: The National Tracheostomy Safety Program (NTSP) [7] and the National Association of Laryngectomy Clubs (NALC) [8]. The NTSP has one e-learning laryngectomy module [7] and NALC offers online information resources, videos and virtual education [8]. These resources predominantly focus on acute laryngectomy management i.e., airway emergency management at the hospital bedside, which is also at the forefront of recent research aiming to develop simulation-based training for urgent laryngectomy care [5]. From a global perspective, laryngectomy commercial companies, Inhealth and ATOS, also offer education and training, however not all components of laryngectomy care are covered or openly available [6].

The resources were included in our recent environmental scan of 49 existing laryngectomy education/training resources for healthcare professionals [6]. This scan concluded that there was no consistent approach to education/training development method, content, delivery or evaluation. Additionally, most of the resources included in the scan had limited patient and public involvement (PPI) at any stage. This highlights a wider power imbalance in which individuals living with a laryngectomy - who universally lose their natural voice and may endure extended periods of profound voicelessness - are afforded minimal opportunity to influence education and training to healthcare professionals. PPI is essential in the design of any health resource: it helps ensure higher quality materials and that the resource will therefore lead to improved patient experience [9]. This likely means that the existing resources included in the environmental scan lack impact for both the healthcare professionals (for example, improved knowledge and skills in laryngectomy care), and patients, (for example, more confidence in community healthcare professionals) alike [6].

### The gap

The findings from this previous work suggest the need for the development of resources specifically for healthcare professionals working in community settings with people with a laryngectomy [6]. Moreover, these resources should be developed collaboratively with patients and healthcare professionals, with a clear implementation and evaluation plan [10].

This gap is being addressed by the CREATOR study (a co-design laryngectomy education and training package for community healthcare professionals), funded through the lead author’s National Institute of Health Research (NIHR) Doctoral-Clinical Academic Fellowship (NIHR 303067). The study’s healthcare professional advisory group and patient and public involvement and engagement (PPIE) members recommended returning “back to basics” by creating an accessible entry point for the training package - such as a poster, infographic, or animation. They emphasised the importance of co-developing the package with both healthcare professionals and PPIE contributors to ensure that the needs of people with a laryngectomy and their families remain central to the training. This collaborative approach aligns with NIHR PPIE standards [9], principles of co-development that engage stakeholders in shaping intervention content and delivery [11], and established intervention design frameworks [12, 13].

### Aims

We describe the development of basic, accessible, entry point resource jointly with people with a laryngectomy. This work is the first step in the co-development of a bespoke education and training package for healthcare professionals working with people with a laryngectomy in the community. Our secondary aim was to reflect the experiences of key project stakeholders including people with a laryngectomy, a graphic designer and an executive from the creative agency.

## Main text

### Developing the concept

The idea for the animation and infographic was based on two stakeholder group discussions on the results of the environmental scan [6]. The two groups were a PPI group and a healthcare professional stakeholder group from the CREATOR study. The PPI group included a range of members including people with a laryngectomy, people who had received other treatment for head and neck cancer and family members from across England and Scotland. In total seven people took part in this PPI group. To note, all patient and public involvement time was reimbursed in accordance with NIHR payment rates. Members of the healthcare professional stakeholder group, five in total, were also from across England and included a range of professional backgrounds including nurses, speech and language therapists and surgeons. Both groups met online via Microsoft Teams for 90 min with discussions facilitated by the lead author. The chat function was enabled to ensure that all those present were able to contribute and the sound-suppression was switched off during the PPI group to ensure that people with a laryngectomy who used alternative communication methods were not ‘cut-off’ by technological settings. The groups initially met separately, as per the principles of experience-based co-design [11], to gather responses from each individual group, before bringing the groups together to share this feedback and work collaboratively on resource development.

Each group (both which had met as part of previous CREATOR project’s research) received a short presentation from the lead author with a statement of the problem the CREATOR project was intending to address and the findings from the environmental scan. This was followed by an open group discussion regarding the results, what these meant clinically, and potential next steps/areas explore. During discussion with both groups, there was overwhelming feedback that a ‘back to basics’ resource needed to be developed in a simple, accessible format as a starting point to help support community-based healthcare professionals working with people with a laryngectomy and their families. Both groups agreed that an animation and/or a simple infographic would be the most suitable way to take this idea forward, with the intention that the final output could form part of the training package developed in the co-design phase of the CREATOR study. They also recognised the need for additional specialist expertise to support the design and development of these materials.

### Collaboration with freelance illustrator and creative agency

One of the PPIE group members had previously worked on some research with a creative agency ‘Creo Comms’ [14] and introduced them to the lead author (LJW). They discussed the project idea with LJW’s employer, South Tyneside and Sunderland NHS Foundation Trust (STSFT). STSFT have an established collaboration with Creo Comms and Sunderland University. This collaboration enables Sunderland University design students to work on an innovative healthcare project as part of their academic training. Following a written brief provided by LJW to Creo Comms, it was agreed that the animation and infographic development was an appropriate student project, a freelance illustrator student (DW) was appointed.

The animation and infographic costs were funded by Head and Neck Charitable Funds at STSFT.

### Development of the animation and infographic

The animation script was co-developed in subsequent sessions by LJW, the STSFT laryngectomy support group (5 people with a laryngectomy, 7 family members), the national association of laryngectomy clubs (2 people with a laryngectomy), CREATOR’s PPI group (7 group members – mix of people with a laryngectomy, people who have had treatment for other head and neck cancers and family members) and CREATOR’s healthcare professional advisory group (mix of professionals from across England with expertise in laryngectomy). These groups were held either virtually or in-person, depending on the preference and geographical location of each group, for example, meetings with the STSFT laryngectomy support group were in-person as the group meets in this way monthly, whereas meetings with CREATOR’s patient and public involvement group were online due to the geographical spread of the group’s participants. Involvement of multiple groups ensured that there was widespread consultation from patients, the public and healthcare professionals regarding the content of the animation and infographic.

LJW met with each group individually to discuss the brief and gather input on the content requirements for the animation and infographic. During these meetings, the scope of the resources was agreed, including the number of sections, level of detail, areas of emphasis, and key messages. Six core sections—anatomy, communication, airway, eating and drinking, treating everyone as an individual, and key messages—were agreed upon, alongside an introduction and a signposting/conclusion section. People with a laryngectomy determined the sequence and key messages, prioritising airway and communication changes over eating and drinking, reflecting what they viewed as most important for healthcare professionals to understand. Decisions regarding length and voice-overs were also made at this stage, with agreement that individuals with a laryngectomy would narrate sections such as communication changes and key messages.

An initial draft of the script was then developed by LJW. This was then reviewed by everyone in each group. Feedback was incorporated into the revised script which was then agreed for use collectively. The script was then reviewed by the creative agency and designer for final sign off.

The student designer developed prototype animation characters following a briefing about the laryngectomy population from LJW. Educational images/diagrams were also sent to the designer following the meetings to help visualise laryngectomy anatomy, products and communication tools. The prototype animation characters were then reviewed by LJW and the supervisory team (JP, LS, DH, VT). Amendments were made to some of the characters to ensure that a range of demographic variables were represented in the animation, ensuring equality, diversity and inclusion e.g., age range, gender and ethnicity of people with a laryngectomy.

The design student initially created draft slides with characters and background scenes for the six key sections in the storyboard. These slides covered one element from each of the six key sections: anatomy, communication, airway, eating and drinking, treating everyone as an individual, and key messages. These were reviewed by all key stakeholders with suggested revisions then shared to be incorporated into the slides. An example of a revision made was a change to the background on the airway safety at home slide. Following this, voice overs were provided for each slide – these were from a mix of people with a laryngectomy and healthcare professionals. A sample of slides with voice-overs were sent for review prior to finalising the animation. In total, the animation underwent four iterations based on feedback from the groups involved prior to agreement on the final version. Iterations to the animation included an additional introductory slide to acknowledge how the animation had been designed, changes to the airway safety animation to make this clearer and more succinct, amendments to the communication slides to make these more interactive. The final animation was eight minutes in length. Key words displayed in writing were incorporated on top of the animated images to support with accessibility and understanding of specific areas, for example, names of communication devices that some people with a laryngectomy use. The finalised animation was then condensed into a one-page infographic using text and characters from the animation to highlight the key messages from each of the six sections included in the animation.

### Animation/Infographic screening and feedback

A hybrid event was held for the animation and infographic screening in February 2025, to ensure everyone involved in the project could be in attendance via their preferred or most suitable method. Six people with a laryngectomy, two family members, three healthcare professionals, the creative agency and designer attended the event alongside the lead author who facilitated the screening. All those in attendance were part of the development group. The animation and infographic were viewed followed by a group discussion seeking attendees’ feedback on each. LJW knew all people with a laryngectomy and if required, discussed any specific communication needs with them to ensure they could contribute fully to the event, for example, using writing or messaging to communicate. The event was scheduled for long enough to ensure that all people with a laryngectomy had the time and opportunity to contribute meaningfully and comfortably.

The reactions to the resources were positive, for example, a group member fed back: *“We both thought the animation was tremendous and would be a very good addition to material available.”* Discussion focussed on the content, length of animation, and further use of the animation beyond the CREATOR study, including potential dissemination strategies, target audiences and ideas for ongoing development and research. Overall people felt that the animation was comprehensive, covering all key elements of living with a laryngectomy, with the freelance illustrator reporting his experiences of working on the development of the animation/infographic: *“I like to be precise when creating informational or educational material*,* but to my astonishment the information on laryngectomy online was very limited and ambiguous. I believe that the work being done here is extremely important for that reason.”* The group felt that as all key elements of living with a laryngectomy, people, such as non-specialist healthcare professionals, would be encouraged to ask more questions. This is further shown by the freelance illustrator’s personal reflections: *“I began this project almost completely ignorant about laryngectomy and can now confidently say that I understand the basics about the procedure and what it means for the patients and those close to them. These are not things that are complicated to grasp but can make all the difference to people’s lives if more people are aware of them. I feel very proud to have been a part of this and hope it continues to help people.”* Attendees felt that the length of the animation was good and the messages in the animation were framed in a positive and non-daunting way, with a key strength being that voices of people with laryngectomies were represented within the animation.

While primarily intended to provide an entry point to the future education and training package for community healthcare professionals, attendees felt that dissemination of the animation should be wide-reaching with suggested target audiences should not be limited to healthcare professionals but also include patients and families and the public [15]. Finally, the group felt that the infographic was a valuable summary of the animation and something which could be easily displayed in health facility waiting rooms or community centres. The group also felt the infographic could be used as a resource by people with a laryngectomy to easily explain what a laryngectomy means to people who aren’t familiar with laryngectomy, such as their friends, family members or members of the public whom they meet.

### Experiences of involvement & impact on stakeholders

This project has had comprehensive and varied involvement from a range of key stakeholders including people with a laryngectomy, industry partners and designers. Feedback was sought from each of the groups to understand their experiences of being involved in the project, any impact it had had and to identify areas for future directions with the animation and infographic. Key stakeholders who had participated in the co-design groups and/or attended the animation screening event were contacted by email by LJW to invite their reflections on the outputs. Two participants with a laryngectomy subsequently requested a video consultation with the lead author to discuss their perspectives in greater depth prior to submitting their written reflections.

Verbatim feedback from people with a laryngectomy included:

Patient partner 1:

*It was a great project to be involved with…it was an open discussion rather than led. The discussion organically evolved to the impact of day-to-day life living with a laryngectomy*,* which personally I think is an important side for people to understand. This is a great back to basics tool not just for health workers but for me to show people I have just met as to what a laryngectomy involves and the changes it makes on voicing*,* smell*,* taste and confidence.*

*Just because I sound different doesn’t make me different. October 2021 was the first time I heard the word Laryngectomy. A Dr visited my bedside in hospital and said this word to me. Needless to say*,* I was in shock and not knowing what this entailed I looked on Google*,* there I found some very basic medical information. There was no information on how my voice would change forever*,* breathing differently*,* altered appearance and changes in taste and smell. The most important thing to me is the social impact which has been the hardest thing to navigate*,* it’s so easy to get sidelined. My new voice which can be difficult to understand and no longer feminine has had every reaction imaginable from empathy to being ignored.*

Patient partner 2:

*I came to this project a laryngectomee having had my operation almost 16 years ago. This is important because some of the issues raises by the group were novel to them but less so to me i.e.*,* I had found “fixes” to some of the issues. I got a sense*,* perhaps incorrectly*,* that this was solely about anatomical change and post care. However*,* this occurs*,* certainly in my case*,* with a change in identity as well as regular periods of depression. This is almost certainly likely to vary depending upon each individual’s circumstances and may be a function of loss*,* grief*,* anxiety*,* age*,* family support*,* lost opportunities etc. It might be useful to signpost*,* if this is within the scope of the project*,* psychological support.*

*Animation has several benefits*:

1. *It is easy to understand*,* pictures are helpful*,* a bit like storytelling.*
2. *It can be “voiced over” in different languages.*
3. *It can be adapted if new information becomes available.*

*From a laryngectomee perspective it was comprehensive and accurately explained. The patient group was very well facilitated*,* this allowed everyone to feel valued and the group remained on-track. All meetings were at a sensible time*,* appropriately spaced and feedback from previous meetings was timely and useful. The use of a voucher as a reward recognised individuals’ input. These all heightened the value of the project.*

*Having participated in previous laryngectomy workshops and groups*,* in my opinion*,* there are members of the laryngectomy population who either don’t want to participate or are unable to do so*, e.g.,* lacking confidence*,* intimidated by groups*,* don’t have access to computer or don’t know how to use them. Also*,* reliable internet access is essential. There is also the real*,* but often overlooked*,* issue of adult illiteracy which is often a barrier to participation. So*,* to some extent*,* the result of the project may be a biased towards those willing and able to participate.*

*In conclusion*,* this was a very enjoyable and worthwhile project*,* and the output will hopefully be a worthwhile tool to all stakeholders.*

Patient partner 3:

*“The Organisation of the Project*

*I joined the team for the CREATOR project as a patient member. The group of patients and carers selected includes a good range of backgrounds and experiences of a laryngectomy*,* including length of time and functional outcomes. The discussions have been directed with skill and patience*,* and it has been rewarding sharing experiences with the other members. I am sure I am not alone in feeling I have been able to contribute as fully and openly as I would have wished.*

#### The animation

*I don’t think I had anticipated the direction of travel of the work would include the production of a resource such as the animation. As President of NALC*,* I have long been aware of the problems for patients and carers when they encounter clinicians unfamiliar with the need of laryngectomees. I have contributed to the training of clinicians at “tracheostomy” days organised by NHS trusts for their staff and others. However*,* the target group for the animation*,* community nurses and others*,* is one that is not easy to reach. I set up the NALC YouTube channel in 2012 which includes short videos*,* many previously available on DVDs. The videos are aimed at different audiences such as nurses*,* first responders and patients and carers. Though there have been more than 100*,*000 videos streamed*,* I don’t think we have managed to reach more than a small fraction of the target audience.*

*The new animation presents key facts and information*,* including patient safety issues*,* in a concise and straightforward way. It is very suitable for viewing on a mobile phone which increases it accessibility. It will surely encourage clinicians who view it to seek further information to assist them in their work yet will also be of value to family members and the public. I think it can be shared with confidence in the knowledge it reflects the priorities of laryngectomy patients themselves.*

#### The future

*It will not be an easy task to ensure that the animation reaches its target audience to such an extent that makes a real difference. There are no obvious systems and structures to facilitate this*,* not least because the patient group is very small. Hopefully clinicians’ professional bodies and patient groups will be able to promote this resource for wide consideration and use.”*

Healthcare professionals involved in the project said:

I think the animation has turned out great!!

I think that the work you are doing will be really beneficial for other professionals and the community in general.

The freelance illustrator on the project said:

It was a genuine pleasure to work on this project and to meet all the people involved. Some of the things I enjoy most about what I do is the variety in the commissions I undertake, being able to learn something new and most importantly, the opportunity to contribute to causes I believe to be worthwhile. When I take on a project, I find it of the upmost importance to research the subject to the best of my ability, learning about the processes involved in having total laryngectomy was eye opening to say the least. I think the messages the patients are trying to get across here are very straight forward. I was shocked to hear about some of the obstacles they had faced that were easily avoidable as well as learning about the potentially dangerous consequences of not being informed on some of the fundamentals of life as a total laryngectomy patient. I think that putting them at the centre of this project will make it a unique and incredibly valuable resource.

The Senior Account Executive at Creo Comms, said:

It was fantastic working with South Tyneside and Sunderland NHS Trust on this project. By involving both patients and student from the outset, we were able to channel the real-life experiences of those who’d experienced a laryngectomy and the creativity and enthusiasm of the student to deliver something which was really impactful. It was a real collaborative effort by Sunderland Creatives Agency, the University of Sunderland and the NHS and we are proud to have supported the project.

From the perspective of the lead author, the development of the animation and infographic represents a significant professional milestone, particularly due to the genuinely collaborative nature of the project and the opportunity to put people with a laryngectomy at the forefront of the work. In reflecting on the process of agreeing the animation content, the lead author has also identified several personal reflections:

*I had to refrain from leading on what I felt was important to include in the animation*,* and allow this conversation to evolve organically*,* with patient partners leading this. As a clinician*,* I felt that it may be important to include equal weighting to the information provided about all the functional changes post-laryngectomy. However*,* all people with a laryngectomy involved did not feel this way and felt that two areas – communication and airway changes - should be given priority for information delivery. As a researcher I asked questions around this and gleaned what was important to include in this information and how participants wanted the information to be delivered. As a clinician I would have likely had different ideas about this*,* and on reflection*,* I can understand why two of the functional areas are higher priority for people living with a laryngectomy. People with a laryngectomy also wanted the key message to be more about treating them as an individual and giving them the time and space to be able to communicate as well as listening to them. This shifts the message away from the physiological and medicalised information*,* which as a clinician is an area I have always included alongside the psychological*,* social and emotional information. However*,* in my role as a researcher within this work*,* I have reflected on how important it is to shift this focus and put the information about the psychological*,* social and emotional impact front and centre*,* rather than providing likely unnecessarily complex detail on the medicalised information*,* which when prioritised has the risk of over-complicating information and therefore losing the key information*,* message and opportunity to increase others knowledge around laryngectomy.*

### Limitations

Although collaborative in nature, there are limitations to this work which are important to consider. The patient partners involved in this research were generally highly engaged, had routine access to digital technology, and possessed the necessary digital literacy and reading skills to participate fully. Consequently, the group may not be fully representative of the broader laryngectomy population, and certain subgroups - particularly those with limited technological access or lower literacy levels - may be underrepresented in this work. Feedback from patient partners was overall extremely positive, and as such, it will be important that broader feedback is sought from a wider range of people with a laryngectomy. Additionally, all authors are UK-based and thus nuances in other countries may not be fully represented in this work. To date, no formal agreement has been made for where and how the animation and infographic will be distributed. However, plans for this are ongoing and will be incorporated into the next phase of this research.

### Next steps

The animation, along with the infographic, will be used at the start of a series of co-design workshops in the CREATOR study to support the development of an education and training package for non-laryngectomy specialist healthcare professionals, to better support people with a laryngectomy accessing care in the community. Whilst it is unknown how many people are living with a laryngectomy globally, the most recent UK-based audits report between 1,196 and 1,216 people living with a laryngectomy [16, 17]. As such, it is anticipated that this research will benefit this relatively small yet highly underserved population by contributing to improved understanding, more consistent care practices, and enhanced support across healthcare settings [3]. Following this, the animation and infographic will made freely available via charities, local support groups and professional organisations and networks. A plan for how to do this will be developed with people with a laryngectomy and healthcare professionals together as part of the co-design workshops. This will enable wide access for all healthcare professionals, the laryngectomy community and public.

## Conclusions

This project has demonstrated the value of stakeholder engagement in research and importance of collaborative working in the development of resources born out of identified gaps from the research. If this project had been done individually or solely with healthcare professionals, the key message of the animation would likely have been different and weighting towards areas that people with a laryngectomy feel are important may well have been under-represented. The perspectives of individuals with lived experience are frequently omitted in the development of healthcare resources due to entrenched power hierarchies in which clinical expertise is prioritised and positioned as superior, while experiential knowledge is not afforded equal legitimacy. This imbalance results in the routine marginalisation of patient voices – which with altered communication can be even more prevalent - and limits the extent to which resources reflect the realities and priorities of those most affected [18]. The co-design process in this study directly countered these dynamics by ensuring that the voices of those most affected shaped both the content and emphasis of the final resource. This work is integral in supporting the development of a comprehensive intervention for community laryngectomy care, specifically incorporating a co-designed education and training package. Beyond this, the animation and infographic are novel ways of disseminating information in head and neck cancer care, with wide application and potential reach beyond the initial intended target audience and may contribute to raising awareness with the public.

## Data Availability

Animation and infographic will be available upon completion of Laura-Jayne Watson’s current research funding via the NIHR.
